# Non-human Primate Papillomaviruses Share Similar Evolutionary Histories and Niche Adaptation as the Human Counterparts

**DOI:** 10.3389/fmicb.2019.02093

**Published:** 2019-09-10

**Authors:** Zigui Chen, Teng Long, Po Yee Wong, Wendy C. S. Ho, Robert D. Burk, Paul K. S. Chan

**Affiliations:** ^1^Department of Microbiology, The Chinese University of Hong Kong, Hong Kong, China; ^2^Centre for Emerging Infectious Diseases, Faculty of Medicine, The Chinese University of Hong Kong, Hong Kong, China; ^3^Department of Pediatrics, Microbiology and Immunology, Epidemiology and Population Health, and Obstetrics, Gynecology and Woman’s Health, Albert Einstein College of Medicine, The Bronx, NY, United States

**Keywords:** primate papillomaviruses, niche adaptation, evolution, divergence time estimate, macaque monkey

## Abstract

Given high genetic diversity of papillomaviruses (PV) and complex scenario of virus-host interaction, the genetic basis underlying the mechanisms of HPV carcinogenicity is not well understood. In an effort to evaluate the origin and evolution of PV pathogenicity, we collected paired oral, perianal, and genital swabs from a wild macaque population. Of the 117 surveyed macaques, 88 (75.2%) were positive for PV DNA in one or more sites, mostly common from genital swabs, followed by oral and perianal sites. All putative macaque PV types phylogenetically clustered into the genera *Alpha*-, *Beta*-, and *Gammapapillomavirus*, with a strong phylogeny-tropism association as observed in HPVs. Using a Bayesian Markov Chain Monte Carlo framework, we demonstrated ancient intra-host divergence of primate PVs in which multiple ancestors had split and adapted to specific host ecosystems at least 41 million years ago, prior to the speciation events of primate host species. Following subsequent divergence and niche adaptation, distinct but phylogenetically related PV types were transmitted to similar host ecosystems by closely related host animals when host speciation occurred, which may explain in part the origin of carcinogenicity of HPV type 16 (HPV16) and *Macaca fascicularis* PV type 3 (MfPV3) that evolved from a most recent common ancestor containing the determinants for cervicovaginal colonization and cervical cancer. The findings identifying evolutionary and biological relatedness between human and non-human primate PVs lay a genetic foundation for research on parasite-host interactions and carcinogenic outcomes, which will prove useful in further study of viral pathogenesis and host specificity.

**STUDY IMPORTANCE:**

To better understand the origin and evolution of PV carcinogenicity associated with cervical cancer, we applied a combination of phylogenetic and bioinformatic analyses to investigate the genetic diversity of macaque papillomaviruses, and estimate divergence times of human and non-human primate PVs. The majority of both human and non-human primate PVs cluster into α-, β-, and γ-PVs, sharing similar evolutionary histories and biological properties to each other. The strong phylogeny-tropism association of primate PVs indicates an important role of niche adaptation and virus-host codivergence shaping the diversity of viral genomics, host specificity, immune exposure, and pathogenic property. Understanding the evolution of the family *Papilloamviridae* in general and the primate papillomaviruses in specific in relevant to virus-host interactions should provide important implications for viral pathogenesis and disease prevention.

## Introduction

*Papillomaviridae* comprised a large family of double-stranded circular DNA viruses of about 8,000 nucleotides in size. Papillomavirus (PV) genome contains three regions, including an upstream regulatory region (URR), an early region of open reading frames (ORFs) (E1, E2, E4, E5, E6, and E7) encoding functional proteins, and a late region of ORFs encoding structural proteins (L1 and L2) (Harden and Munger, 2017). PVs infect the cutaneous and mucosal epithelia of a large spectrum of vertebrates including mammals, reptiles, and rodents even fishes (Rector and Van Ranst, 2013). To date, more than 200 types of human PVs (HPVs) and 160 types of animal PVs have been fully characterized (Van Doorslaer et al., 2018). The clinical consequence of PV infections ranges from completely asymptomatic to benign warts or even malignant neoplasia (Graham, 2017). For example, HPV type 16 (HPV16) and HPV18 contribute to over 70% of cervical cancers, the 4th most common cancer in women worldwide (Bravo et al., 2010). The majority of HPVs are classified into 3 genera: *Alphapapillomavirus* (α-PV), *Betapapillomavirus* (β-PV), and *Gammapapillomavirus* (γ-PV) (de Villiers et al., 2004; Bernard et al., 2010). All genital carcinogenic HPVs are members of the genus α-PV, while only a limited number of β-HPVs have been associated with squamous cell cancer of the skin, particularly in subjects with epidermodysplasia verruciformis (EV). The pathogenic role of γ-HPVs is unclear, while skin swab and oral cavity harbor abundant known and novel β- and γ-HPVs (Nindl et al., 2007; Bottalico et al., 2011; Wong et al., 2018).

Besides of carcinogenic HPVs, a large set of non-human primate PVs within the species *Alphapapillomavirus* 12 (α12) isolated from the cervicovaginal region of macaques have been associated with cervical cancers (Wood et al., 2007; Chen et al., 2009). The species α12 (oncogenic macaques PVs) are phylogenetic related to the species α9 (oncogenic HPV16) but distantly separated from some β- and γ-PV types that were also isolated from macaque animals (e.g., MfPV1 within the species β1), indicating that there is an evolutionary relatedness of papillomavirus carcinogenicity while they may not strictly follow the virus-host codivergence (Chen et al., 2018a). Codivergence is a process of reciprocal and cross-adaptive genetic changes, which means that the evolutionary history of pathogens should reflect that of its host counterparts in divergence times as well as phylogenetic histories (Hafner and Nadler, 1988; Woolhouse et al., 2002). For papillomaviruses, however, the prior adaptation of pathogens to various ecological niches may explain the conflicts between pathogens and hosts phylogenies (Chan et al., 1992; Bravo et al., 2010; Shah et al., 2010; Sharp and Simmonds, 2011; Chen et al., 2018a), which was also documented for other viruses, such as polyomaviruses, herpesviruses and some retrovirus genera (McGeoch et al., 2006; Niewiadomska and Gifford, 2013; Buck et al., 2016).

Papillomaviruses are ancient DNA viruses that have co-evolved with their hosts over millions of years, resulting in a high level of diversity at host specificity, tissue tropism, viral genetics, prevalence and pathogenesis (Shah et al., 2010; Van Doorslaer et al., 2017). The importance of evolutionary dynamics in driving PV divergence has been increasingly recognized recently, which largely facilitates our understanding on the genetic basis underlying the mechanisms of viral carcinogenicity (Bernard et al., 2006; Bravo et al., 2010). However, the knowledge on the origin and evolution of PV oncogenicity remain scarce. In this study, we collected oral, perianal and genital swabs from a wild macaque population (*Macaca mulatta*) inhabiting a protected nature reserve in Hong Kong. Genetic diversity and evolutionary relationship among macaque PVs as well as among HPVs were characterized, with divergence time estimated using Bayesian molecular clock model to trace the divergence of primate PVs within niche-specific clades. The findings identifying biological and evolutional relatedness between human and non-human primate PVs lay a genetic foundation for research on parasite-host interactions and carcinogenic outcomes, which will prove useful in further study of viral pathogenesis and host specificity.

## Materials and Methods

### Macaque Sample Collection, DNA Extraction, and Papillomavirus Genotyping

Between August and December 2016, paired swab samples from oral, perianal and genital (vaginal sites from female macaques, and penial sites from male macaques) sites of 117 non-captive wild rhesus macaques (*M. mulatta*), including 84 females and 33 males, were collected from Kam Shan Country Park, Hong Kong, China (Chen et al., 2018b). All animals appeared healthy according to the body condition and the temperature measured by veterinarians. Swab samples were collected in 2 ml specimen transport medium (STM) [Hank’s BSS (10×), 5% Bovine Albumin (BA), Gentamycin (4 mg/ml), Pen/Strept (50,000 μg/ml), Fungizone (1 mg/ml), and NaHCO3 7.5%] when the animals were anesthetized for the veterinary and/or contraceptive treatment and transported to the laboratory in an ice box cooler within 2 h.

The total DNA was extracted using Qiagen DNA Mini Kit (Qiagen, United States) with 200 μl STM suspension and eluted in 200 μl elution buffer (pH 8.0) for PCR-based papillomavirus genotyping. Two recently developed next-generation sequencing (NGS) amplicon assays targeting the consensus regions of PV L1 open reading frame of the full spectrum of primate papillomaviruses including α-, β-, and γ-PVs were used (Fonseca et al., 2015; Agalliu et al., 2016). Successful amplicons, with a pair of unique 12-bp barcodes introduced by forward and reverse primers, were pooled and sequenced on an Illumina Miseq (Illumina, United States) at the Weil Cornell Medicine Genomics Resourses Core Facility, New York, NY, United States, using paired-end 150 bp reads.

Following the demultiplexing, the paired short reads passing the quality filter (≥Q20 and ≥50-bp) were merged into single reads using FLASh v1.2.11 (Magoc and Salzberg, 2011) and blasted against a papillomavirus reference database using UPARSE software (Edgar, 2013). An operation taxonomic unit (OTU) count table was created using a 95% identity threshold (Fonseca et al., 2015; Wong et al., 2018), with each OTU assigned for PV type based on sequence homology to the reference database: if OTUs hitting the reference database had ≥90% identities to a characterized PV type, they represented known viruses; those with 60–89% identities were regarded as “uncharacterized” types and assigned with a unique identity. A PV type was considered positive if the reads were ≥50. For each PCR amplification, multiple negative controls and random repeats were set in order to measure the false positive and minimize the potential contamination.

### Characterization of Macaque Papillomavirus Complete Genomes

The complete genomes of *M. mulatta* papillomaviruses (MmPV2 to 7) were characterized using virus-enriched shotgun sequencing, as previously reported (Chen et al., 2018a; Long et al., 2018). Whole viral genomes were validated using type-specific PCR amplification in three or four overlapping fragments and Sanger sequencing using a primer walking strategy. ORFs were predicted using Geneious v9 and searched for homology using a BLASTX against the closely related HPV types.

### Phylogenetic Analyses and Tree Construction

Phylogenetic analysis was based on the concatenated nucleotide sequence alignments of papillomavirus genes. The codon sequences from each ORF were aligned based on the corresponding amino acid sequences aligned using MUSCLE v3.8.31 (Edgar, 2004). Maximum likelihood (ML) trees were constructed using RAxML MPI v8.2.3 (Stamatakis, 2006) with optimized parameters. Data were bootstrap resampled 1,000 times. MrBayes v3.1.2 (Ronquist and Huelsenbeck, 2003) with 100,000,000 cycles for the Markov chain Monte Carlo (MCMC) algorithm was used to generate Bayesian trees. A 25% discarded burn-in was set to eliminate iterations at the beginning of the MCMC run. The average standard deviation of split frequencies was checked to confirm the independent analyses approach stationarity when the convergence diagram ModelTest v3.7 (Posada and Crandall, 1998) was used to identify the best evolutionary model; the identified general time reversible (GTR) model was set for among-site rate variation and allowed substitution rates of aligned sequences to be different. The CIPRES Science Gateway (Miller et al., 2010) was accessed to facilitate RAxML and MrBayes high-performance computation. Phylogenetic trees were visualized using FigTree v1.4.4 and Ape v5.1 (Paradis and Schliep, 2019).

### Divergence Time Estimation

We used a Bayesian MCMC method implemented by BEAST v2.4.5 (Drummond and Rambaut, 2007) and the previously published PV evolutionary rates (Rector et al., 2007) to estimate the divergence time of primate PVs from their most recent common ancestors (MRCAs). Separated estimations were performed for each of the genera α-, β- and γ-PV since primate PVs, taken together, do not follow strict virus-host codivergence. Three tree priors (coalescent constant population, Yule model, and coalescent Bayesian skyline) and two molecular clock models [strict mutation rate and relaxed uncorrelated lognormal distribution (UCLD)] were pre-tested, with a combination of Yule and relaxed UCLD of molecular clock model showing the lowest Akaike’s information criterion for MCMC samples (AICM) value (Baele et al., 2012). The concatenated codon sequence partitions of six ORFs (E6, E7, E1, E2, L2, and L1) with variable rates of substitution over time as previously published (Rector et al., 2007): E6 (2.39 E-08 substitutions per site per year, 95% confidence interval of 1.70 – 3.26 E-08), E7 (1.44 E-08, 0.97 – 2.00 E-08), E1 (1.76 E-08, 1.20 – 2.31 E-08), E2 (2.11 E-08, 1.52 – 2.81 E-08), L2 (2.13 E-08, 1.46 – 2.76 E-08), and L1 (1.84 E-08, 1.27 – 2.35 E-08). In order to calibrate the divergence times, we introduced three time points in the α-PV tree, with assumptions of codivergence histories between primate PVs and their hosts: (1) the split between α-PVs and *Saimiri sciureus* PV 1/2/3 (SscPV1/2/3, within the genus *Dyoomikronpapillomavirus*) at 49 (95% CI of 41 – 58) million years ago (mya) matching the divergence between Old World and New World monkey ancestors (Perez et al., 2013), (2) the separation between the species α12 and α9 at 28 (25 – 31) mya matching the speciation between hominin and macaque ancestors (Gibbs et al., 2007), and (3) the node between HPV13 and *Pan paniscus* PV 1 (PpPV1) at 7 (6 – 8) mya matching the split between hominin and chimpanzee ancestors (Patterson et al., 2006). For β- and γ-PV trees, the calibration time point (s) was set between macaque PVs and their closest HPV relatives. The MCMC analysis was run for 100,000,000 steps, with subsampling every 10,000 generations. A discarded burn-in of the first 10% steps was set to refine trees and log-files for further analysis. Effective sample sizes (ESS) of all parameters are >300 (*Alphapapillomavirus* tree) and >2000 (HPV variant trees of each type), indicating that all Bayesian chains were well sampled and have converged. The divergence times of non-human primate PVs partially sequenced by MY and FAP were estimated using the L1 substitution rate and the evolution models as mentioned above.

## Results

### Prevalence of PV Infection in Rhesus Monkeys

In order to understand PV infection in a macaque population, we used two amplicon assays to detect the presence of DNA of a broad spectrum of PV types including α-, β-, and γ-PVs from paired oral, perianal and genital (vaginal sites for females, and penial sites for males) swabs from 117 wild rhesus macaques (*M. mulatta*). A total of 88 macaques (75.2%) had detectable PV DNA in one or more body sites, with similar infection rate between female (76.2%, 64/84) and male animals (72.7%, 24/33) (*p* = 0.8123). As expected, genital swabs had the highest infection rate (55.6%, 65/117), followed by oral (35.9%, 42/117), and perianal (29.9%, 35/117) sites (Table 1).

**TABLE 1:** Distribution of papillomavirus infection in a surveyed macaque population.

**Site**	**No. (%) of samples tested**		**No. (%) of PV-positive samples within each genus^a^**			**No. of unique PV types detected within each genus**				**Cumulative no. (%) of PV infection detected within each genus**			
	**Total**	**PV-positive**	**α**	**β**	**γ**	**Total**	**α**	**β**	**γ**	**Total**	**α**	**β**	**γ**
Genital	117	65 (55.6)	56 (86.2)	1 (1.5)	21 (32.3)	52	32	1	19	198	159 (80.3)	1 (0.5)	38 (19.2)
Perianal	117	35 (29.9)	18 (51.4)	1 (2.9)	18 (51.4)	36	17	1	18	72	43 (59.7)	1 (1.4)	28 (38.9)
Oral	117	42 (35.9)	3 (7.1)	4 (9.5)	38 (90.5)	28	2	1	25	72	3 (4.2)	4 (5.6)	65 (90.3)
Total	351	142 (40.5)	77 (54.2)	6 (4.2)	77 (54.2)	72	32	1	39	342	205 (59.9)	6 (1.8)	131 (38.3)

*^*a*^Samples with PV infection from >1 genus were counted for each genus.*

Cross body site infection was common in the surveyed macaques (Supplementary Table S1). At least 31 (36.9%) female and 12 (36.4%) male animals contained PV viruses in two or three body sites. Altogether, 10 animals (8.5%) were PV DNA positive for all three body sites.

### Distribution of PV Genera Between Body Sites

**FIGURE 1:**
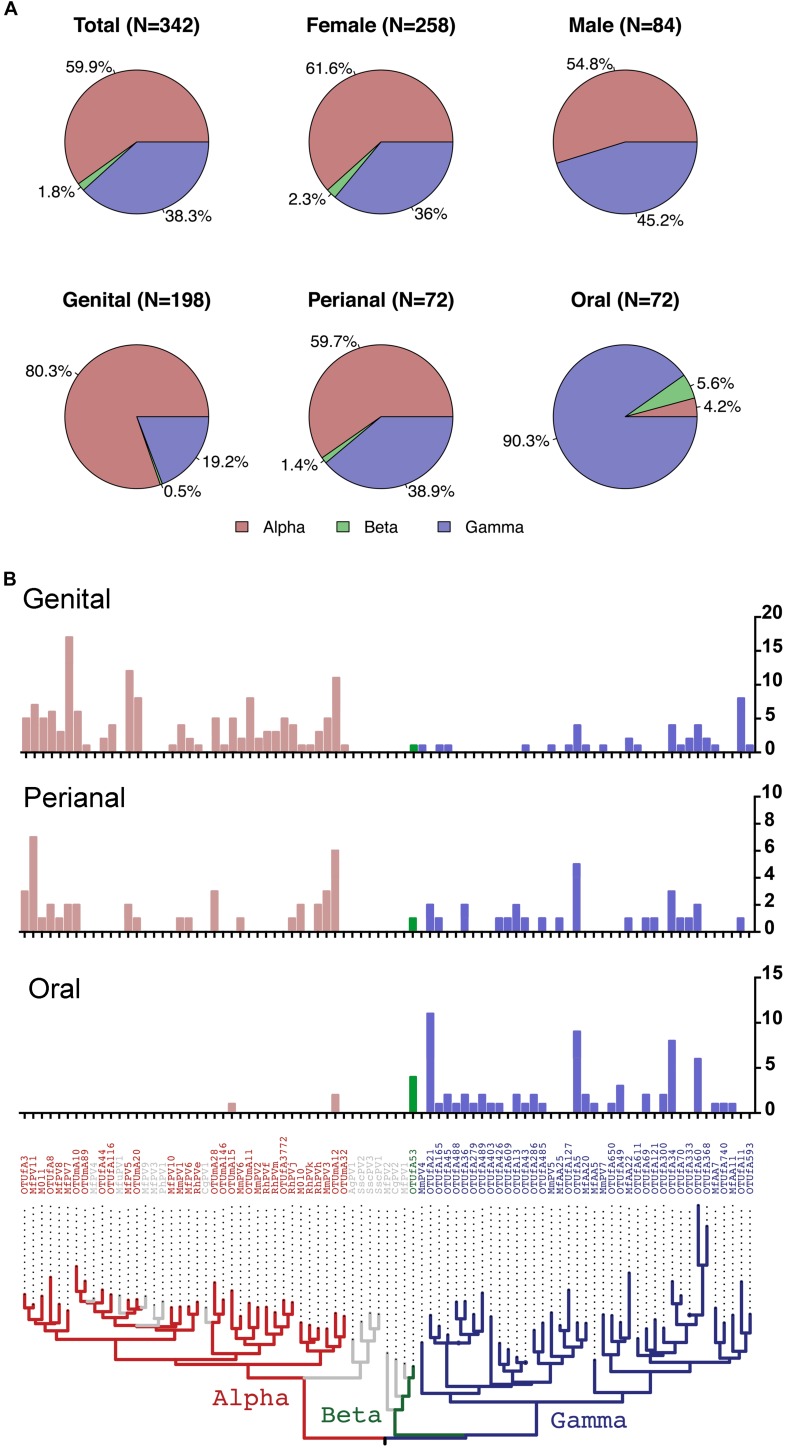
Distribution of macaque PV genera and types in different body sites. **(A)** Pie charts showing the proportion of cumulative macaque PV infection between genera by gender and body sites. **(B)** Distribution of macaque PV infections in genital, anal and oral sites. The *y*-axis of the bar charts represents numbers of infections of each type. The phylogenetic tree on the bottom of the panel was inferred from previously reported macaque PV complete genomes and partial L1 sequences detected in this study. Animals with co-infections were counted more than once. Colors in the phylogenetic tree indicate different genera including Alpha-PV in red, Beta-PV in green, and Gamma-PV in blue.

On the basis of partial L1 sequence information, a total of 72 putative macaque PV types were observed and phylogenetically clustered into the genera α-, β-, and γ-PV. Among PV-containing samples (*n* = 142), 77 (54.2%) were infected with at least one α-PV type, 6 (6.2%) with a β-PV type, and 77 (54.2%) with a γ-PV type (Table 1). Interestingly, we found significant difference in the distribution of PV genera between samples isolated from different body sites. For instance, 56 out of 65 PV-positive genital swabs (86.2%) had α-PV infection compared to 7.1% (3/42) of oral infection; whereas 90.5% (38/42) of oral infection were γ-PV compared to 32.3% (21/65) of genital infection (*p* < 0.001). In consistence with the tropism of body site between macaque PV genera, all 32 putative α-PV types were able to be detected in genital samples while only 2 types in oral swabs. We found 25 and 19 γ-PV types in oral and genital swabs, respectively, with 8 types in both sites (Table 1 and Supplementary Table S2). When the total number of PV infection (abundance) was considered, the tropism was also significant: genital samples had 80.3% of α-PV infections and 19.2% of γ-PV infections when compared to 4.2% of α-PV infections and 90.3% of γ-PV infections in oral cavity (*p* < 0.001) (Figure 1A and Table 1). The anal swabs had similar proportion of α- and γ-PV infections, inferred from the infected samples (51.4% vs. 51.4%), the unique PV types (17 vs. 18), and the PV abundance (59.7% vs. 38.9%) (Figure 1B and Table 1).

The β-PV was rare in the surveyed macaques. Only one type was detected that infected 5 animals (4 oral samples, 1 anal sample and 1 vaginal sample) (Supplementary Table S1). MfPV7, a member type within the α12 species, was the most common mucosal type found in 17 genital samples and 2 perianal samples (Supplementary Table S2). A number of α-PV types, including OTUmA12 (*n* = 19), OTUfA3 (*n* = 17), MfPV5 (*n* = 14), MfPV11 (*n* = 14) and MmPV6 (*n* = 11), commonly found in genital and/or perianal sites were absent in oral cavity. OTUfA21 (*n* = 11), OTUfA5 (*n* = 9) and OTUfA34 (*n* = 8) phylogenetically clustered into the γ-PV genus, however, were more common in oral samples, although they might infect perianal and/or genital sites occasionally (Supplementary Table S2).

Multiple-PV type infection in a same sample was common. 54.2% of PV-containing samples (77/142) were infected with at least two or more types, mostly common in genital swabs (63.1%, 41/65), followed by perianal (54.3%, 19/35), and oral (40.5%, 17/42) samples.

Eighteen macaque animals shared same PV type(s) between body sites. Identical types were found more commonly in perianal-genital pairs (*n* = 12), followed by oral-perianal pairs (*n* = 6), and oral-genital pairs (*n* = 2). OTUmA12 and OTUmA28, two α-PV types, were mostly detected in 4 and 3 perianal-genital pairs, respectively. It is worth to note that infection of a same PV type crossing body sites could be from different sources but the close anatomic locations between genital and perianal areas likely facilitated the viral transmission from one body site to another more easily.

### Detection of Novel Macaque PV Genomes

A BLAST search using partial L1 sequences against GenBank/NCBI and our PV database identified 25 potential novel α-PV types, 1 novel β-PV type, and 39 novel γ-PV types (Supplementary Table S2). Among them, 6 α-PV types (RhPVe, f, h, j, k, and m) and 6 γ-PV types (MfAA5, 7, 11, 20, 22, and 25) have been previously reported as partial MY and FAP sequences obtained from genital tissues (Chan et al., 1997) and skin swabs (Antonsson and Hansson, 2002), respectively.

The complete genomes of six macaque papillomaviruses (*M. mulatta* PV type 2, 3, 4, 5, 6, and 7) isolated from the surveyed animals have been previously characterized by the authors’ group. As shown in Supplementary Figure S1 of the predicted genomic structure, the viral genomes range from 7,292 bp to 7,935 bp in size, with five putative early genes (E6, E7, E1, E2, and E4), two late genes (L2 and L1) and an URR between the L1 and E6 genes. Although a small ORF (53 amino acids) between the E2 and L2 genes of MmPV2 and MmPV6 showed relatively high homology to the 3’-terminus of HPV6 E5a (33.9%), this gene warrants further characterization due to short sequence length. The L1 nucleotide sequence pairwise comparison and phylogenetic analysis indicated that these novel PV types shared less than 73% identities to the closest HPV relatives and well clustered into the genera α-PV (MmPV-2, -3, and -6) and γ-PV (MmPV-4, -5, and -7). Since all surveyed animals appeared healthy according to the body condition and the temperature measured by veterinarians, the pathogenic potential of these macaque PV types remains to be established.

### Niche Adaptation of Non-human Primate PVs

**FIGURE 2:**
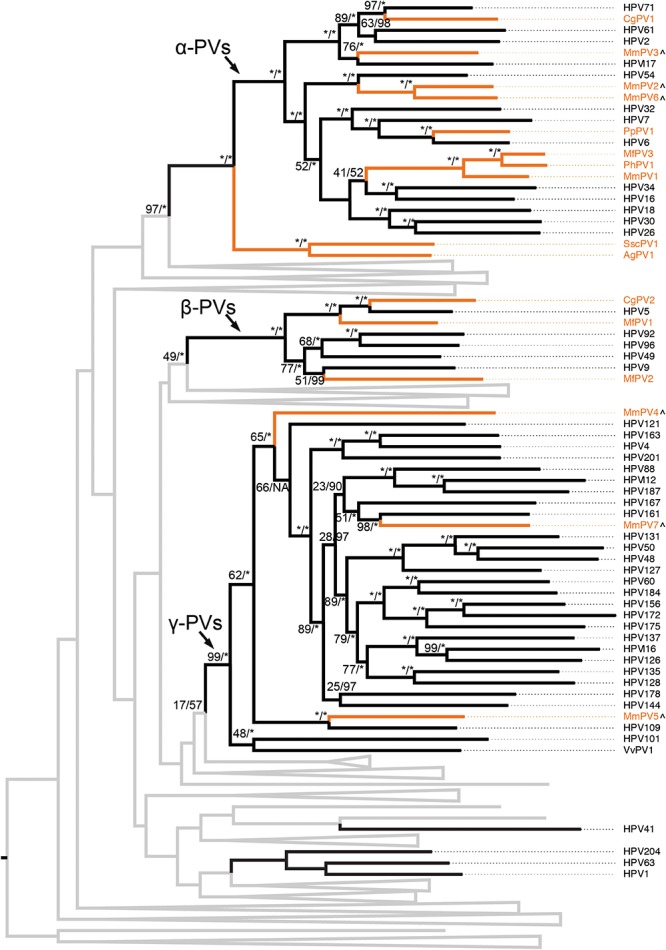
Phylogeny of primate papillomaviruses. A maximum likelihood (ML) phylogenetic tree was constructed based on the concatenated nucleotide sequence alignment of 6 ORFs (E6-E7-E1-E2-L2-L1) of 145 papillomavirus types representing 136 species infecting unique host species (see PV list with hosts in Supplementary Table S3). The majority of analyzed primate papillomaviruses clustered into three distinct genera, Alpha- Beta- and Gamma-PV. The branches representing non-human primate papillomaviruses are highlighted in orange. Non-primate animal papillomaviruses branches are collapsed in gray lines (see comprehensive tree in Supplementary Figure S2). Numbers on the branches indicate support indices of maximum likelihood bootstrap percentages using RAxML and Bayesian credibility value percentage using MrBayes. 100% agreement is denoted by an asterisk (^∗^). Novel macaque PV complete genomes characterized in this study are highlighted with a (∧) besides of the taxa labels.

The evolutional history of the family *Papillomaviridae* as well as the primate PVs specifically was examined using the concatenated nucleotide sequence alignments of six ORFs (E6-E7-E1-E2- L2-L1) of 145 PV types representing 136 species from unique host species (Supplementary Table S3). As expected, the majority of human and non-human primate PVs clustered into three genera, α-, β-, and γ-PVs (Figure 2 and Supplementary Figure S2). Similar as the human counterparts, non-human primate PVs infecting a same host species usually did not cluster together but phylogenetically formed clades corresponding to host ecological sites where the viruses were predominantly isolated. For example, macaque PVs within the species α12 (e.g., MmPV1) isolated from cervicovaginal region of rhesus and cynomolgus monkeys shared a MRCA with genital oncogenic HPV types within the species α9 (e.g., HPV16) and α11 (e.g., HPV34) but were distantly related to MmPV4/5/7 (within the genus γ-PV) from the same macaque species. The data suggest that primate PVs, as a whole clade, did not follow strict virus-host codivergence and likely have evolved from multiple ancestors adapting to specific host ecosystems before the speciation events of host species. The phylogenetic topology of primate PVs was well supported by the trees inferred from either early genes (E1-E2) or late genes (L2-L1), although evolutionary incongruences for limited clades were observed inferred from different genes (Supplementary Figure S3).

**FIGURE 3:**
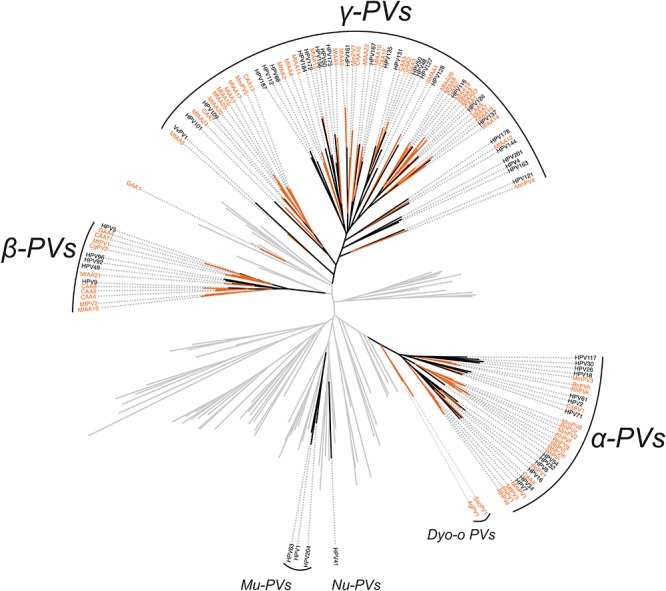
Phylogeny of primate papillomaviruses inferred from partial L1 sequences from previous publications and the current study. A maximum likelihood phylogenetic tree was constructed using RAxML based on the nucleotide sequence alignment of L1 gene of 145 characterized papillomaviruses and 53 putative non-human primate papillomaviruses (see PV list in Supplementary Table S4). The majority of primate papillomaviruses clustered into three distinct clades, termly Alpha- Beta- and Gamma-PV genera. The branches representing non-human primate papillomaviruses are highlighted in orange. Non-primate animal papillomaviruses branches are collapsed in gray lines.

In order to validate the phylogenetic distribution of non-human primate PVs in association with host ecosystem tropism, we searched NCBI/GenBank and assessed 53 partial sequences previously characterized from a wide range of apes and monkeys (Supplementary Table S4). Nearly all non-human primate PV sequences (except for GAA1 from the skin region of a gorilla) clustered into the genera α-, β- and γ-PV, sharing the same tree topology as that of human counterparts (Figure 3). Furthermore, these viruses formed clades by anatomic sites where they were isolated: RhPVs (RhPVc, e-k, m) reported as partial MY sequences isolated from genital tracts of *M. mulatta* entirely cluster into the genus α-PV (Chan et al., 1997); in contrast, sequences obtained from skin swabs of *P. paniscus* (CAA1 to 13), *Gorilla gorilla* (GAA2 to 4), *Ateles fusciceps* (SMAA1), and *Macaca fascicularis* (MfAA1 to 26) phylogenetically located within β- and γ-PV genera (Antonsson and Hansson, 2002).

### Ancient Intra-Host Divergence of Primate PVs Prior to the Host Speciation

**FIGURE 4:**
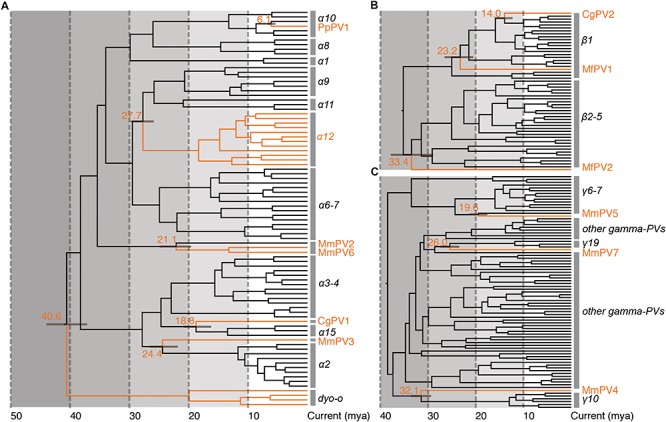
Divergence time estimation of primate papillomaviruses to their most recent common ancestors (MRCAs). The times were estimated separately for each genus **(A)** Alpha-PVs, **(B)** Beta-PVs, and **(C)** Gamma-PVs. A Bayesian MCMC method was used to estimate the divergence time as described in section “Materials and Methods.” The branch lengths are proportional to the divergence times in million year ago (mya), with branches in orange referring to non-human primate papillomaviruses split from their MRCAs with their closest HPV relatives. The bar on the nodes represent the 95% highest posterior density (HPD) interval for the divergence times (see details in Supplementary Figures S4–S6, respectively).

Following Fahrenholz’s rule for strict virus-host codivergence (Hafner and Nadler, 1988), the evolutionary history of a pathogen should mirror that of its host, both in phylogenetic topology and divergence time. In order to estimate the split time between human and non-human primate PVs from their MRCAs, we applied a Bayesian statistical algorithm employing previously reported PV evolution rate and a combination of relaxed lognormal molecular clock and Yule population models. The divergence times were further calibrated by providing host fossil data in the trees, with assumption of codivergence histories between primate PVs and their hosts within specific phylogenetic clades. We observed a deep separation between Dyoomikron-PVs (represented by *S. sciureus* PV 1/2/3) and α-PVs split from a MRCA approximately 41 mya (95% highest posterior density, HPD, 37 – 44 mya) (Figure 4A and Table 2), coinciding with the time frame of host speciation between New World and Old World monkeys. Most of macaque PVs diverged from the MRCAs with their closest HPV relatives around 14 – 33 mya, the time span roughly overlapping the speciation between macaque and human ancestors that occurred approximately 25 – 31 mya (Figure 4 and Supplementary Figures S4–S6). Chimpanzee PVs (e.g., PpPV1) likely shared a MRCA with HPVs (e.g., HPV13) around 6 mya; this was also the time period that most of distinct HPV types emerged. The divergence times were further confirmed by the estimation including all α-, β- and γ-non-human primate PVs partially sequenced by MY and FAP (Supplementary Figure S7).

**TABLE 2:** Divergence time estimation of Alphapapillomavirus and Dyoomikronpapillomavirus types.

**Calibration**	**Clock model**	**Tree prior**	**AICM value**	**AICM difference**	**Log marginal likelihood**	**Alpha vs. Dyoomega (mean)**	**95% HPD interval**	**Alpha 9/ll vs. Alpha 12 (mean)**	**95% HPD interval**	**MRCA (million years ago, mya)**							
										**Alpha 2 vs. MmPV3 (mean)**	**95% HPD interval**	**Alpha l5 vs. CgPVl (mean)**	**95% HPD interval**	**MmPV2/6 vs. HPV54**	**95% HPD Interval**	**HPV13 vs. PpPVl (mean)**	**95% HPD Interval**
3 Cali.	Relaxed	Bayesian	712342	464	–355596.55	40.7	[37.4, 44.3]	27.5	[25.7, 29.6]	24.5	[21.9, 26.8]	18.7	[16.6, 21.2]	22.2	[19.4, 25.3]	6.0	[5.4, 6.7]
3 Cali.	Relaxed	Constant	712333	455	–355627.96	41.0	[37.5, 44.3]	27.8	[25.7, 29.8]	24.5	[22.0, 26.9]	18.8	[16.7, 21.0]	22.1	[19.4, 24.9]	6.1	[5.4, 6.8]
**3 Cali**.^#^	**Relaxed**	**Yule**	**711878**	–	**−55346.34**	**40.6**	**[37.1, 44.0]**	**27.7**	**[25.9, 29.8]**	**24.4**	**[21.9, 26.6]**	**18.8**	**[16.2, 20.9]**	**22.1**	**[19.6, 24.9]**	**6.1**	**[5.4, 6.7]**
3 Cali.	Strict	Bayesian	713733	1855	–56795.11	36.3	[33.6, 38.8]	26.3	[24.4, 27.9]	21.8	[20.2, 23.5]	15.9	[14.8, 17.2]	18.8	[17.4, 20.4]	5.3	[4.9, 5.8]
3 Cali.	Strict	Constant	713776	1898	–356831.68	36.4	[33.9, 39.1]	26.3	[24.6, 28.3]	21.7	[20.2, 23.6]	15.9	[14.6, 17.1]	18.9	[14.6, 17.1]	5.3	[4.9, 5.8]
3 Cali.	Strict	Yule	713196	1318	–356545.10	36.2	[33.8, 38.9]	26.2	[26.6, 30.3]	21.7	[20.1, 23.3]	15.8	[14.7, 17.2]	18.8	[17.2, 20.2]	5.3	[4.9, 5.8]

*^#^The best models determined by the AICM test are highlighted in bold.*

**FIGURE 5:**
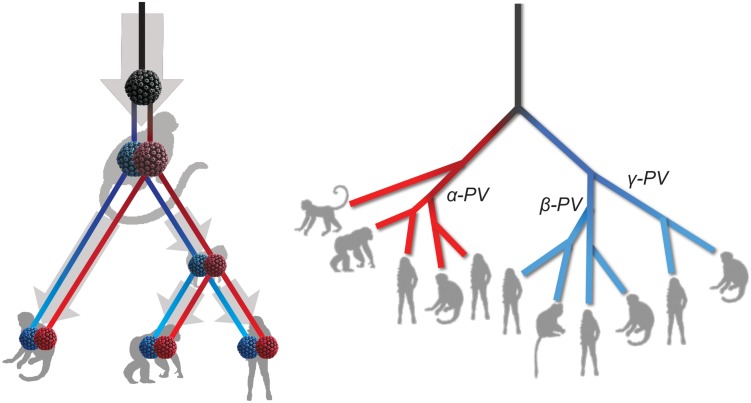
Schematic model of ancient intrahost viral divergence followed by virus-host codivergence in primate papillomaviruses. The **left panel** depicts the model of host niche adaptation of primate papillomavirus ancestors followed by subsequent speciation of host species. Idealized cartoon tree on the **right panel** represents the expected primate PV phylogenic topology.

The tree topologies and divergence times support a model of ancient intra-host divergence of primate PVs in which multiple viral ancestors had split and adapted to specific host ecosystems (e.g., mucosal or cutaneous sites) within an ancestral host animal lineage (e.g., the MRCA of primate animals), prior to the speciation events of host species (Figure 5). Following periods of subsequent divergence and niche adaptation (e.g., different types of epithelial cells), distinct but phylogenetically related PV types were transmitted to similar host ecosystems by closely related host animals when the host speciation occurred. Intra-host viral divergence may occur at multiple time periods and/or host niche locations, resulting in the radiation observed in the extant primate PV tree where viruses sort by tissue tropism and/or biological property but not host species. The model of ancient viral divergence coupled to niche adaptation may explain in part the clustering of α9/11/12 (including HPV16, HPV34, and MfPV3) that evolved from a common ancestor colonizing the cervicovaginal regions of human and macaque ancestor and containing determinants associated with cancer.

## Discussion

In this study, we surveyed a broad spectrum of α-, β-, and γ-PV infection from paired oral, perianal and genital swab samples collected from 117 non-captive healthy rhesus macaques using Next-Gen sequencing amplicon assays. Overall, high infection prevalence and genetic diversity of macaque PVs were detected. All identified macaque PV sequences clustered into the genera α-, β-, and γ-PV, with similar phylogenetic topologies as that of HPVs. Significant tissue tropism of macaque PVs were observed; the majority of PV types isolated from genital regions phylogenetically clustered into the genus α-PV, whereas the oral cavity harbored more γ-PVs. In consideration of low mutation rate of PV evolution and rare interspecies transmission, the close relationship between macaque PVs and HPVs isolated from similar anatomic sites (e.g., mucosal or cutaneous sites) and/or sharing similar pathogenic properties (e.g., α12 and α9 species associated with cancer) suggests a model of ancient divergence of primate PV ancestors adapting to specific host ecosystems prior to the host speciation. This phylogenetic topology was supported by a number of previously reported PV sequences from a wide range of apes and monkeys exclusively clustering into the genus α-, β-, and γ-PVs, in accordance to host anatomic sites where these viruses were originally isolated (Van Ranst et al., 1991; Chan et al., 1997; Antonsson and Hansson, 2002). Although a number of macaque PV types can be detected in multiple anatomic sites of the surveyed animals, making the actual tissue-tropism of primate PVs tentative, the self-transmission of viruses between the close anatomic locations (e.g., perianal and genital areas) should be a factor that could be ignored due to the poor sanitation of monkeys.

Using Bayesian molecular clock models, we estimated ancient intra-host divergence of primate PV ancestors within an ancestral host animal lineage occurred at least 41 million years ago, the era prior to the speciation between New World and Old World monkeys (Perez et al., 2013). Following subsequent divergence and niche adaptation, the speciation events of host animals (e.g., the split between macaques and humans) might isolate viruses with similar biological properties and/or niches into distinct but phylogenetic related clades (e.g., α9/11/12). This scenario may explain the close clustering of oncogenic human and non-human primate PVs (e.g., MfPV3 and HPV16) sharing a most recent common ancestor that contained the determinants for cervicovaginal colonization and cervical cancer. Interestingly, our unpublished data indicates that MfPV3 E6 can significantly degrade host p53 protein. It has been widely reported that high-risk HPV types (e.g., HPV16) can bind and degrade human p53 protein, a tumor suppressor gene targeted by viral E6 protein (Schiffman et al., 2016). The data indicates that certain types of macaque PVs may carry carcinogenic property similar as high-risk HPV types do; the close phylogenetic relationship between α12 and α9 species supports evolutionary adaptation of biological functions between human and non-human primate PVs from their common ancestors.

Virus-host codivergence was generally responsible for the large evolutionary distances between PVs of hosts belonging to different order and/or class (e.g., mammal vs. feline). However, this term does not explain the distribution of primate PVs infecting a single host species into different phylogenetic clades. Ancient intra-host viral divergence undergoing slow genomic mutations in respond to selection pressure by host immune responses and viral genetic drift may have properties beneficial for virus strategies, such as improved infectivity, regulation and pathogenesis within specific host niches. For example, all α-PVs but not β-/γ-PVs contain a short region in length of 300 – 500 bp between E2 and L2 genes, including an E5 ORF sharing high homology between genital high-risk HPVs and MfPVs (Bravo and Alonso, 2004; Chen et al., 2009). The E5 protein has been reported to be important in host-cell transformation (DiMaio and Mattoon, 2001). Niche adaptation is a slow and long-term progress, restricted by ecological and host environment as well as molecular interplaying between virus and host regulatory proteins (Shadan and Villarreal, 1993; Bravo and Felez-Sanchez, 2015). Subsequent speciation events of the host animals were able to isolate virus ancestors within niche-specific clades into different populations, with or without phenotypic differences. The bottleneck of virus transmission between human and non-human primate hosts formed all observed macaque PVs, for example, into distinct clades (e.g., the species α12) excluding any of HPV types, and vice versa, with mirrored phylogenetic topologies and divergence times between hosts and parasites.

Diversification of PV genomes independent from host speciation can occur in non-primate animals harboring many PV types belonging to different genera, such as bovine, canine and feline PVs. At least 27 distinct BPV types have been characterized and were phylogenetically assigned into five genera including Delta- (BPV1, 2, 13 and 14), Xi- (BPV3, 4, 6, 9, 10, 11, 12, 15, 17, 20, 24, 26, and 04AC14), Episilon- (BPV5, 8, and 25), Dyoxi- (BPV7, 19, 21, and ujs21015), and Dyokappa-PV (BPV16, 18 and NY8385) (Rector and Van Ranst, 2013; Van Doorslaer et al., 2018). Bovine PVs have been reported to be associated with mucocutaneous papillomas and neoplasia of the bladder and the upper alimentary tract (Gil da Costa et al., 2017). Interestingly, except for the genus Dyoxi-PV, the rest four genera also contained the majority of PV types isolated from artiodactylous animals, such as *Ovis aries* PVs, *Camelus dromedaries* PVs, and *Cervus elaphus* PVs, suggesting an ancient intra-host viral divergence history occurred in the ancestors of artiodactylous PVs. Following virus-host codivergence within specific host ecosystems, artiodactylous PVs infecting a single host species might form distinct clade that was phylogenetically related to viruses from other closely related host species. We found the similar evolutionary pattern in canine and feline PVs that distributed into the genera Chi-, Lambda- and Tau-PV, although these viruses were mainly isolated from the oral cavity and their pathogenesis warrants further investigation.

It is unclear whether viral transmission occurred between human and non-human primate ancestors. Although there is no report of any HPV DNA being detected in an animal or of any animal PV DNA in a human, the extreme species restriction is probably exclusively operative in primate PVs. Given close cutaneous or mucosal contacts between hominid and macaque ancestors in the early stage of host speciation, host interbreeding and switching via horizontal transmission may be responsible in part for a recent split of MmPV5 and CgPV2 from their closest HPV relatives, as estimated around 13 – 14 mya.

Non-human primates share various behavioral, physiological and genetic similarities with humans, making monkey PVs as ideal animal models to understand the origin and evolution of HPV carcinogenicity. A number of macaque PV types within the species α12 isolated from mucosal lesions of rhesus and cynomolgus macaques have been associated with cervical neoplasia (Wood et al., 2007). The evolutionary relatedness between α12 and α9 species highlights macaque PVs serving as a unique and highly relevant preclinical model that minimizes limitations of mucosal tropism, viral transcription and genomic factors for the study of HPV persistence and oncogenesis (Campo, 2002; Hu et al., 2017).

One of the limitations of this study is the short of cytological and/or histological information to define the potential pathogenicity of macaque PVs in the surveyed macaque population. The most predominant genital macaque PV was MfPV7 found in 19 animals (16%), a member type of the species α12 containing MfPV3 associated with cervical cancer. However, the oncogenic potential of MfPV7 and/or other closely related types within the species α12 is not clearly known. Although the L1-based amplicon sequencing assays were able to detect a broad spectrum of primate PVs, the implementation of whole genome metagenomic sequencing may provide a deep genomic coverage to further explore viral mutation and diversity (Pastrana et al., 2018; Tirosh et al., 2018). We only sampled a single macaque species inhabiting a protected nature reserve. It is possible that an increased diversification of macaque PV types, particular the β-PVs could be observed in populations from different geographic locations and macaque species. To our knowledge, however, this is the most comprehensive study of primate PV genomic heterogeneity and evolution by including all up-to-date non-human primate PV complete genomes and partial sequences, which strengthens the model of ancient intrahost divergence of primate PVs.

## Conclusion

In conclusion, this study establishes a comprehensive theoretical framework on the evolution of primate PVs, suggesting an ancient intrahost viral divergence model upon niche adaptation followed by virus-host codivergence. Our model provides a genetic foundation to better understand the origin and evolution of HPV pathogenesis and carcinogenicity associated with cervical cancer, and the underlying contribution of host niche adaptation to viral fitness. The oncogenic outcomes of human and non-human primate PVs imply an evolutionary convergence of virus-host interaction involving complex interaction between host and pathogens in association with different genotypes, phylotypes and phenotypes.

## Data Availability

Accession numbers for the sequences determined in this study are available in GenBank under the accession numbers MG837557, MG837558, MG837559, MH745747, MH745748, and MH745749.

## Ethics Statement

The animal use protocol has been approved by the Agricultural and Fishery Department and presented to the Animal Welfare Advisory Group (AWAG). All experiments were performed in accordance with the relevant guidelines and regulations.

## Author Contributions

All authors were involved in the conception and design of the study, and approved the final manuscript. ZC, PC, and RB contributed to the study design and supervision. TL, PW, and WH processed the samples and performed the sequencing. ZC and TL contributed to the data analysis and wrote the original draft of the manuscript. PC and RB reviewed and edited the manuscript.

## Conflict of Interest Statement

The authors declare that the research was conducted in the absence of any commercial or financial relationships that could be construed as a potential conflict of interest.

## Supplementary Material

FIGURE S1 Genomic structure of six *Macaca mulatta* papillomavirus complete genomes.

FIGURE S2 A maximum likelihood (ML) phylogenetic tree of papillomaviruses. The tree was constructed using RAxML, based on the concatenated nucleotide sequence alignment of 6 open reading frames (ORFs) (E6-E7-E1-E2-L2-L1) of 145 papillomavirus types representing 136 species and unique host species (see PV list in Supplementary Table S3, column of “Selected type”). The number on the nodes indicate maximum likelihood bootstrap percentages. The main clades containing the majority of primate papillomavirus species are highlighted in gray.

FIGURE S3 Phylogenetic incongruence of papillomaviruses. ML phylogenetic trees were constructed using the concatenated nucleotide sequence alignment of early genes (E1–E2) and late genes (L2–L1) of 145 papillomavirus types representing 136 species and unique host species (see PV list in Supplementary Table S3). The branches in orange represent non-primate papillomaviruses. Non-primate animal papillomaviruses are collapsed in gray. Numbers on the branches indicate support indices of maximum likelihood bootstrap percentages using RAxML, and Bayesian credibility value percentage using MrBayes. 100% agreement is denoted by an asterisk (^∗^).

FIGURE S4 Divergence time estimation of Alphapapillomaviruses and Dyoomikronpapillomaviruses to their most recent common ancestors (MRCAs). A Bayesian MCMC method was used to estimate the divergence time of Alphapapillomariruses and Dyoomikronpapillomaviruses from their most recent common ancestors. The branch lengths are proportional to the divergence times. The branches in orange refer to non-human primate papillomaviruses. The number on the nodes are the mean estimated divergence time in million year ago (mya). The bar on the nodes represent the 95% highest posterior density (HPD) interval for the divergence times.

FIGURE S5 Divergence time estimation of Betapapillomaviruses to their most recent common ancestors. A Bayesian MCMC method was used to estimate the divergence time of Betapapillomaviruses from their most recent common ancestors. The branch lengths are proportional to the divergence times. The branches in orange refer to non-human primate papillomaviruses. The number on the nodes are the mean estimated divergence time in mya. The bar on the nodes represent the 95% HPD interval for the divergence times.

FIGURE S6 Divergence time estimation of Gammapapillomaviruses to their most recent common ancestors. A Bayesian MCMC method was used to estimate the divergence time of Gammapapillomaviruses from their most recent common ancestors. The branch lengths are proportional to the divergence times. The branches in orange refer to non-human primate papillomaviruses. The number on the nodes are the mean estimated divergence time in mya. The bar on the nodes represent the 95% HPD interval for the divergence times.

FIGURE S7 Divergence time estimation of Alphapapillomaviruses and Dyoomikronpapillomaviruses (A), Betapapillomaviruses (B), and Gammapapillomaviruses (C) inferred from partial L1 sequences by MY and FAP. A Bayesian MCMC method was used to estimate the divergence time of non-human primate PVs and HPVs from their most recent common ancestors. The branch lengths are proportional to the divergence times. The branches in orange refer to non-human primate papillomaviruses. The number on the nodes are the mean estimated divergence time in mya. The bar on the nodes represent the 95% HPD interval for the divergence times.

TABLE S1 Papillomavirus infection in a surveyed macaque individuals.

TABLE S2 Macaque papillomavirus types infecting in a surveyed macaque population.

TABLE S3 List of papillomavirus complete genomes used in this study.

TABLE S4 List of partial L1 gene of papillomavirus types analyzed in this study.
