# Supplementary figures and images for: Non-human Primate Papillomaviruses Share Similar Evolutionary Histories and Niche Adaptation as the Human Counterparts
Source: Front Microbiol. 2019 Sep 10;10:2093. doi: 10.3389/fmicb.2019.02093 (PMC6747053; doi:10.3389/fmicb.2019.02093)

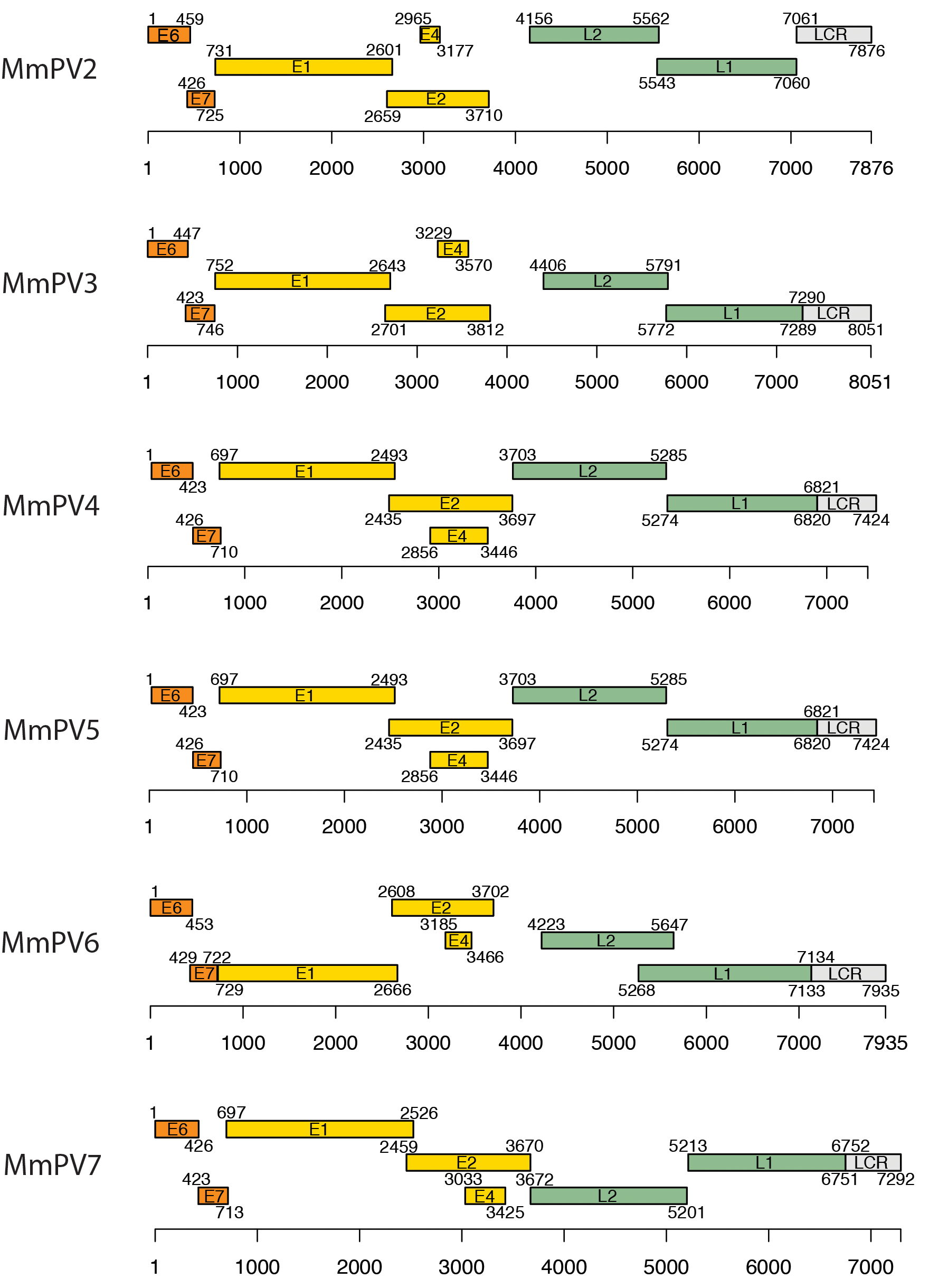

Supplement: FIGURE S1 — Genomic structure of six Macaca mulatta papillomavirus complete genomes. [file Image_1.TIF]

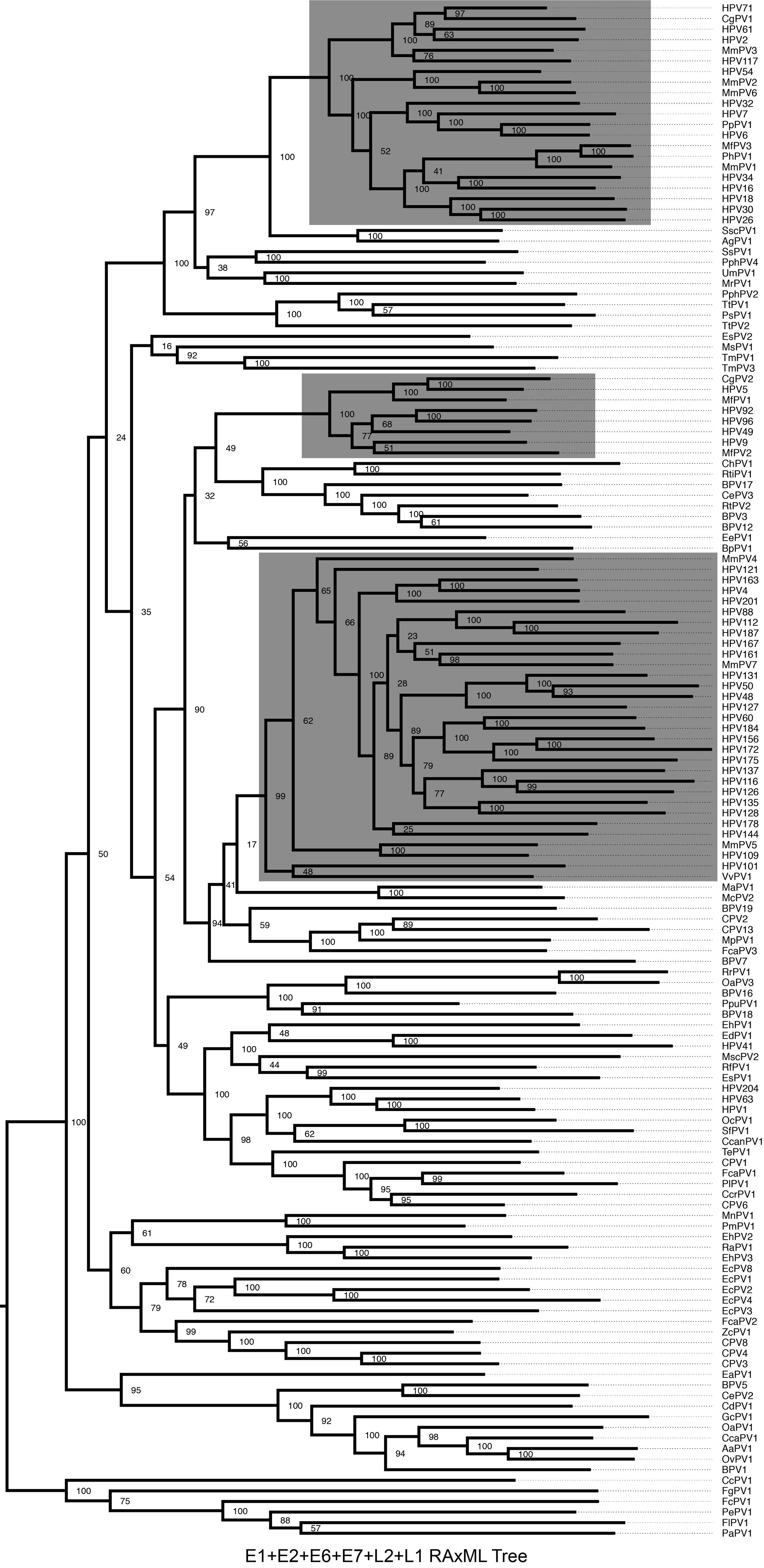

Supplement: FIGURE S2 — A maximum likelihood (ML) phylogenetic tree of papillomaviruses. The tree was constructed using RAxML, based on the concatenated nucleotide sequence alignment of 6 open reading frames (ORFs) (E6-E7-E1-E2-L2-L1) of 145 papillomavirus types representing 136 species and unique host species (see PV list in Supplementary Table S3, column of “Selected type”). The number on the nodes indicate maximum likelihood bootstrap percentages. The main clades containing the majority of primate papillomavirus species are highlighted in gray. [file Image_2.TIF]

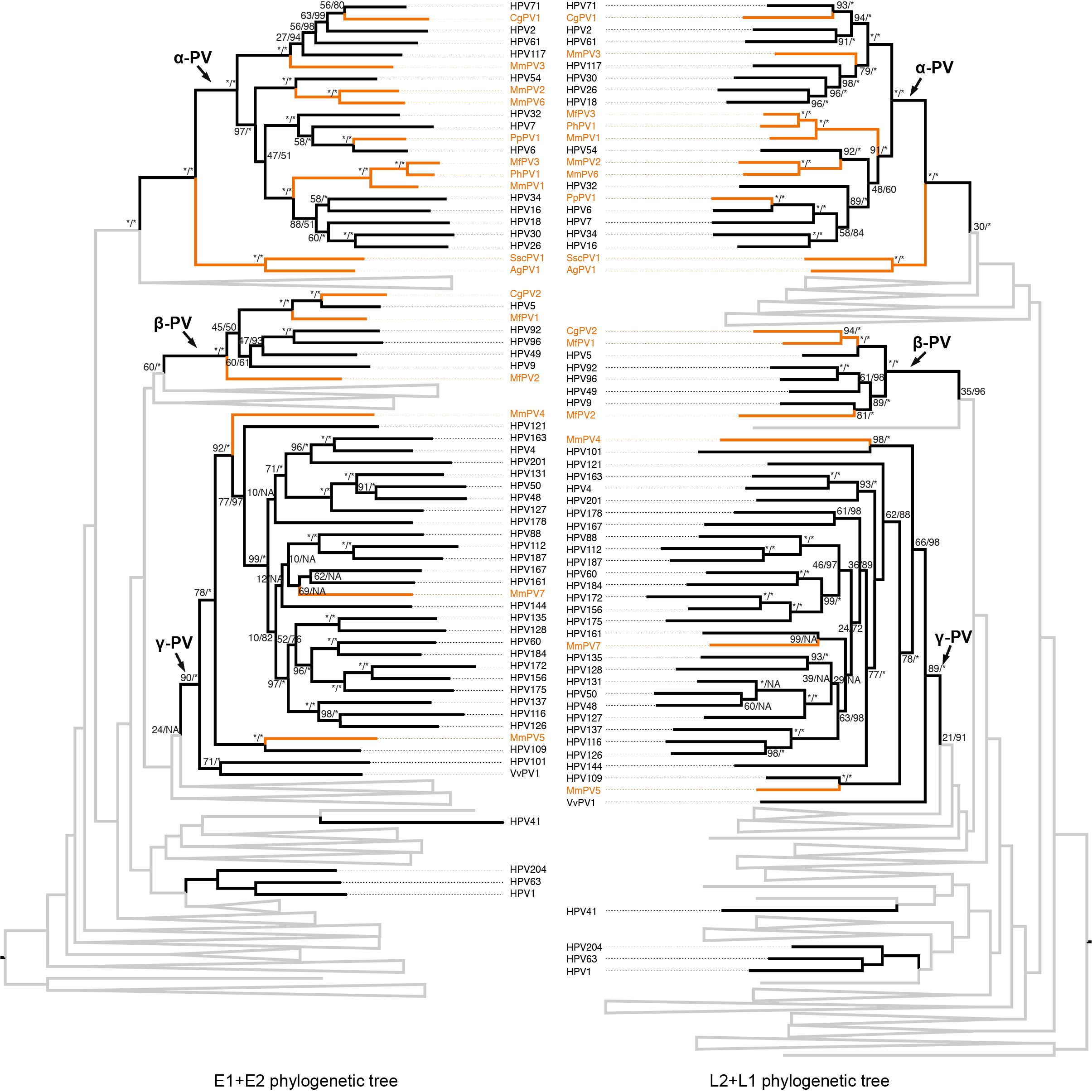

Supplement: FIGURE S3 — Phylogenetic incongruence of papillomaviruses. ML phylogenetic trees were constructed using the concatenated nucleotide sequence alignment of early genes (E1–E2) and late genes (L2–L1) of 145 papillomavirus types representing 136 species and unique host species (see PV list in Supplementary Table S3). The branches in orange represent non-primate papillomaviruses. Non-primate animal papillomaviruses are collapsed in gray. Numbers on the branches indicate support indices of maximum likelihood bootstrap percentages using RAxML, and Bayesian credibility value percentage using MrBayes. 100% agreement is denoted by an asterisk (∗). [file Image_3.TIF]

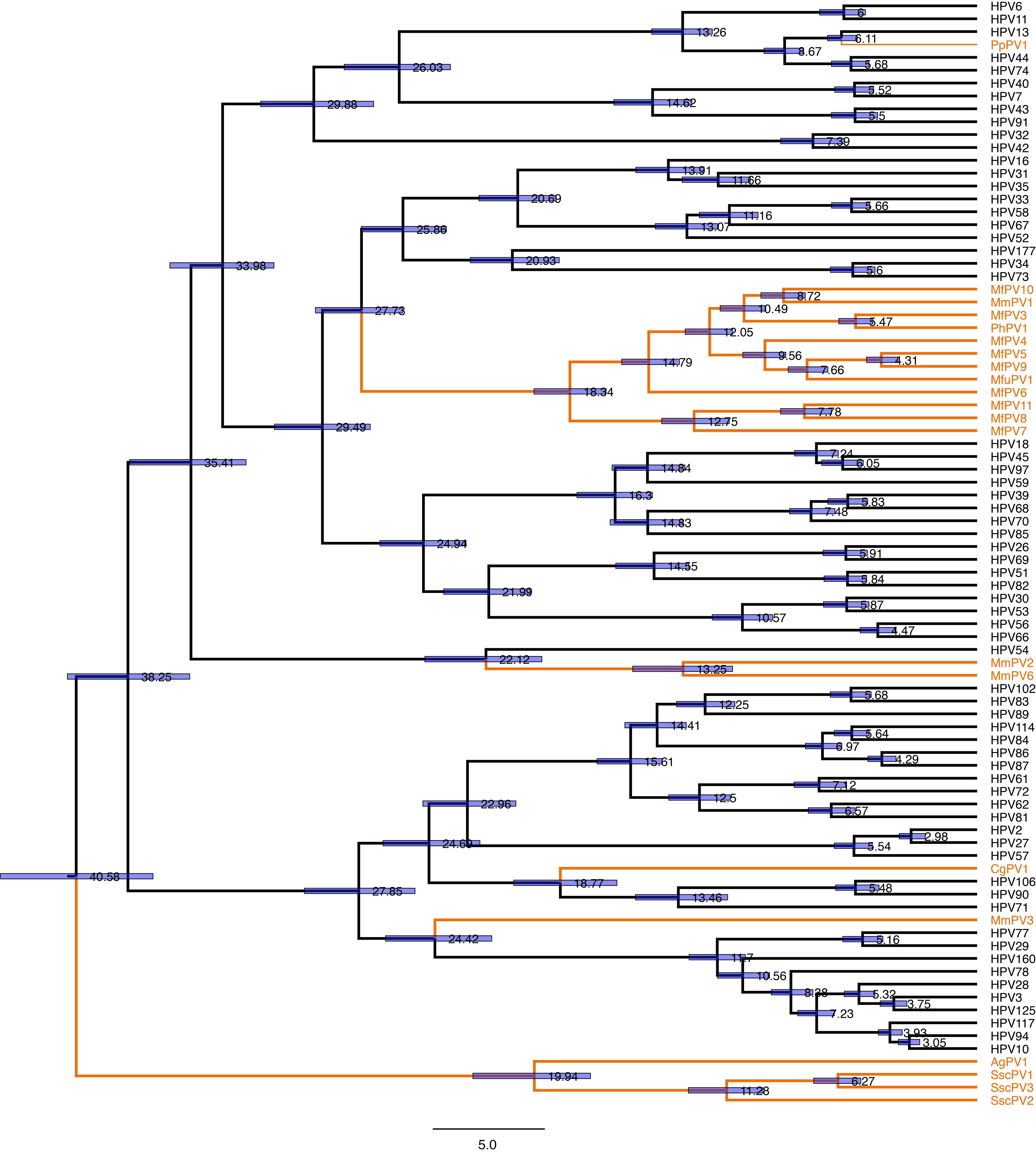

Supplement: FIGURE S4 — Divergence time estimation of Alphapapillomaviruses and Dyoomikronpapillomaviruses to their most recent common ancestors (MRCAs). A Bayesian MCMC method was used to estimate the divergence time of Alphapapillomariruses and Dyoomikronpapillomaviruses from their most recent common ancestors. The branch lengths are proportional to the divergence times. The branches in orange refer to non-human primate papillomaviruses. The number on the nodes are the mean estimated divergence time in million year ago (mya). The bar on the nodes represent the 95% highest posterior density (HPD) interval for the divergence times. [file Image_4.TIF]

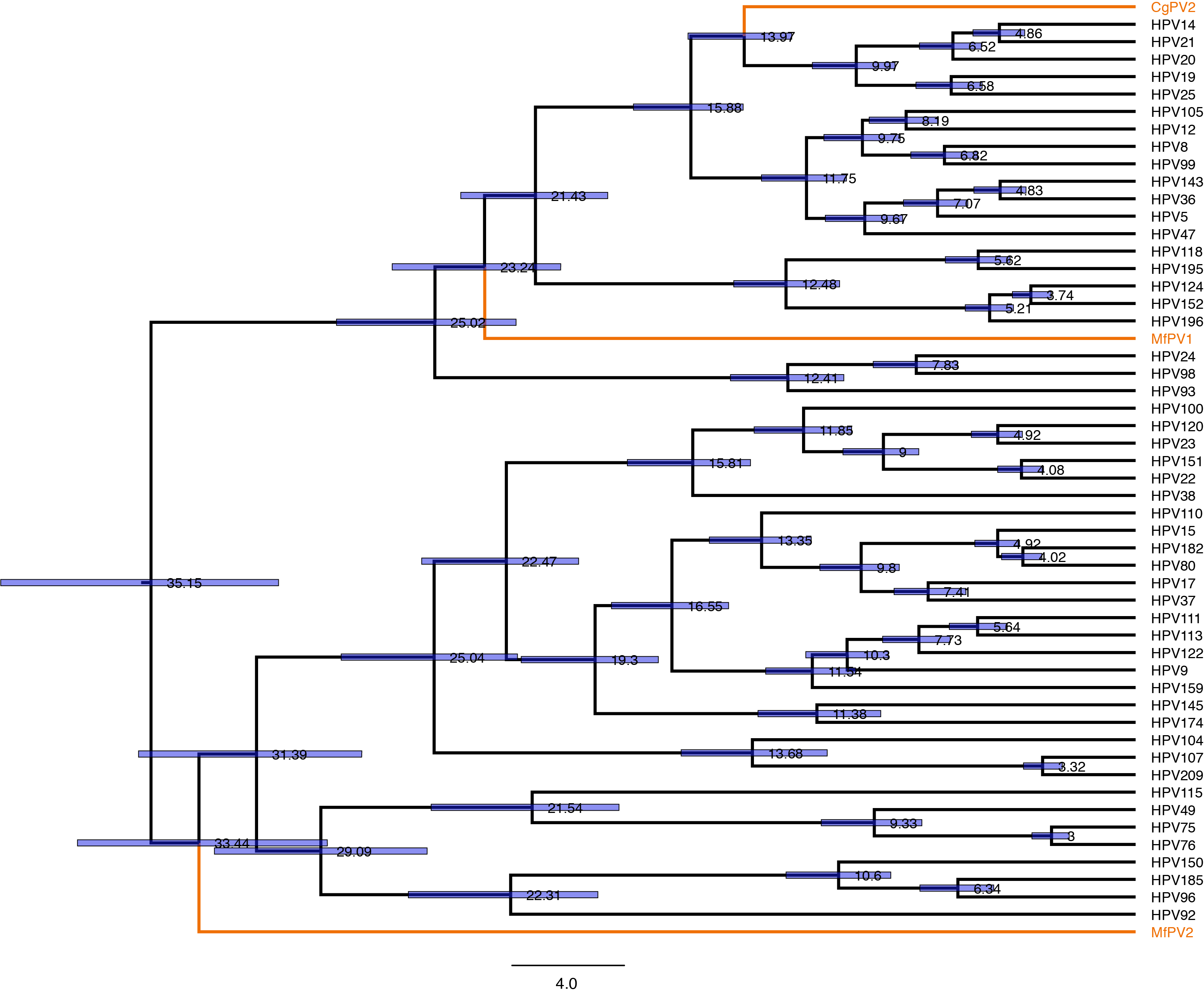

Supplement: FIGURE S5 — Divergence time estimation of Betapapillomaviruses to their most recent common ancestors. A Bayesian MCMC method was used to estimate the divergence time of Betapapillomaviruses from their most recent common ancestors. The branch lengths are proportional to the divergence times. The branches in orange refer to non-human primate papillomaviruses. The number on the nodes are the mean estimated divergence time in mya. The bar on the nodes represent the 95% HPD interval for the divergence times. [file Image_5.TIF]

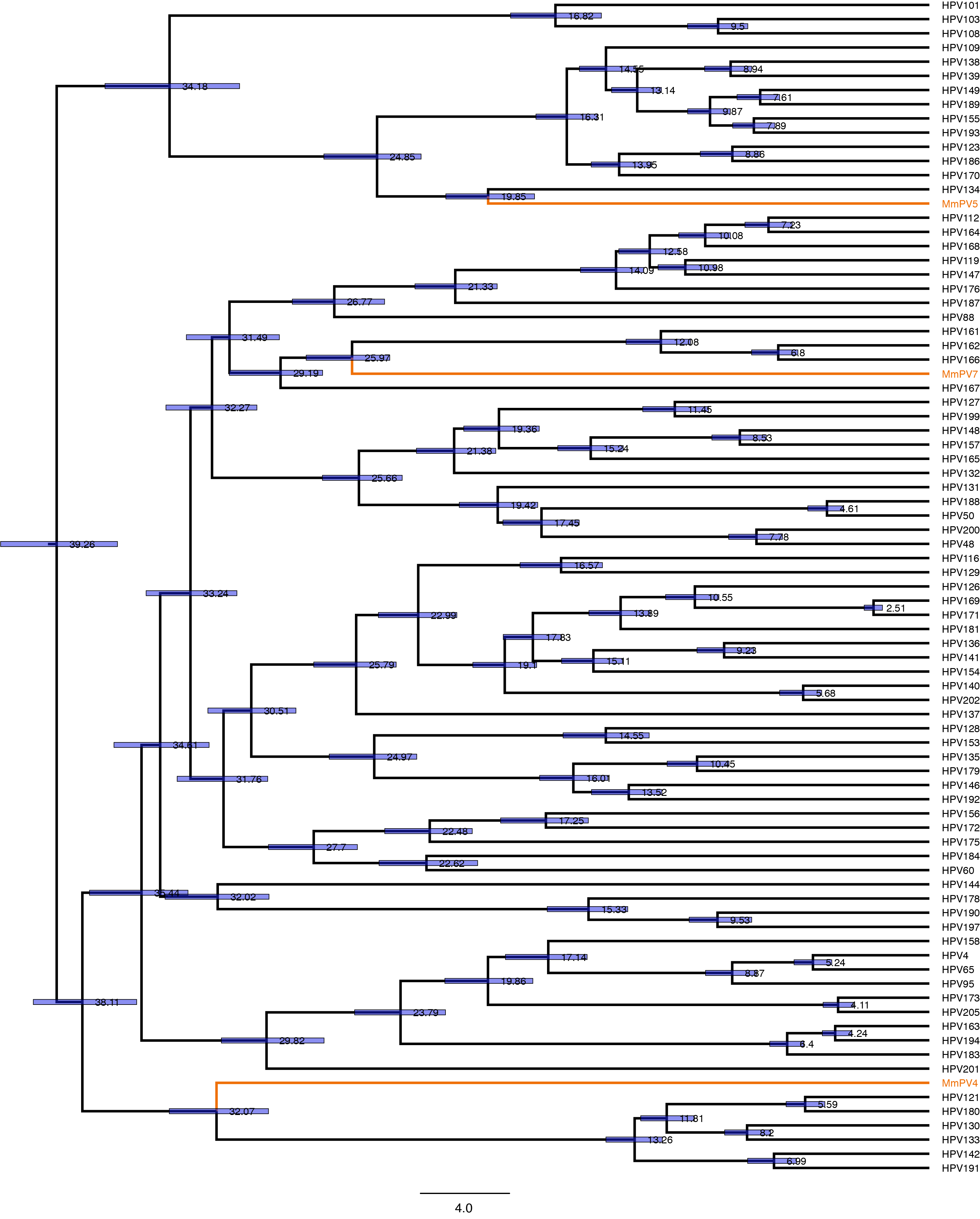

Supplement: FIGURE S6 — Divergence time estimation of Gammapapillomaviruses to their most recent common ancestors. A Bayesian MCMC method was used to estimate the divergence time of Gammapapillomaviruses from their most recent common ancestors. The branch lengths are proportional to the divergence times. The branches in orange refer to non-human primate papillomaviruses. The number on the nodes are the mean estimated divergence time in mya. The bar on the nodes represent the 95% HPD interval for the divergence times. [file Image_6.TIF]

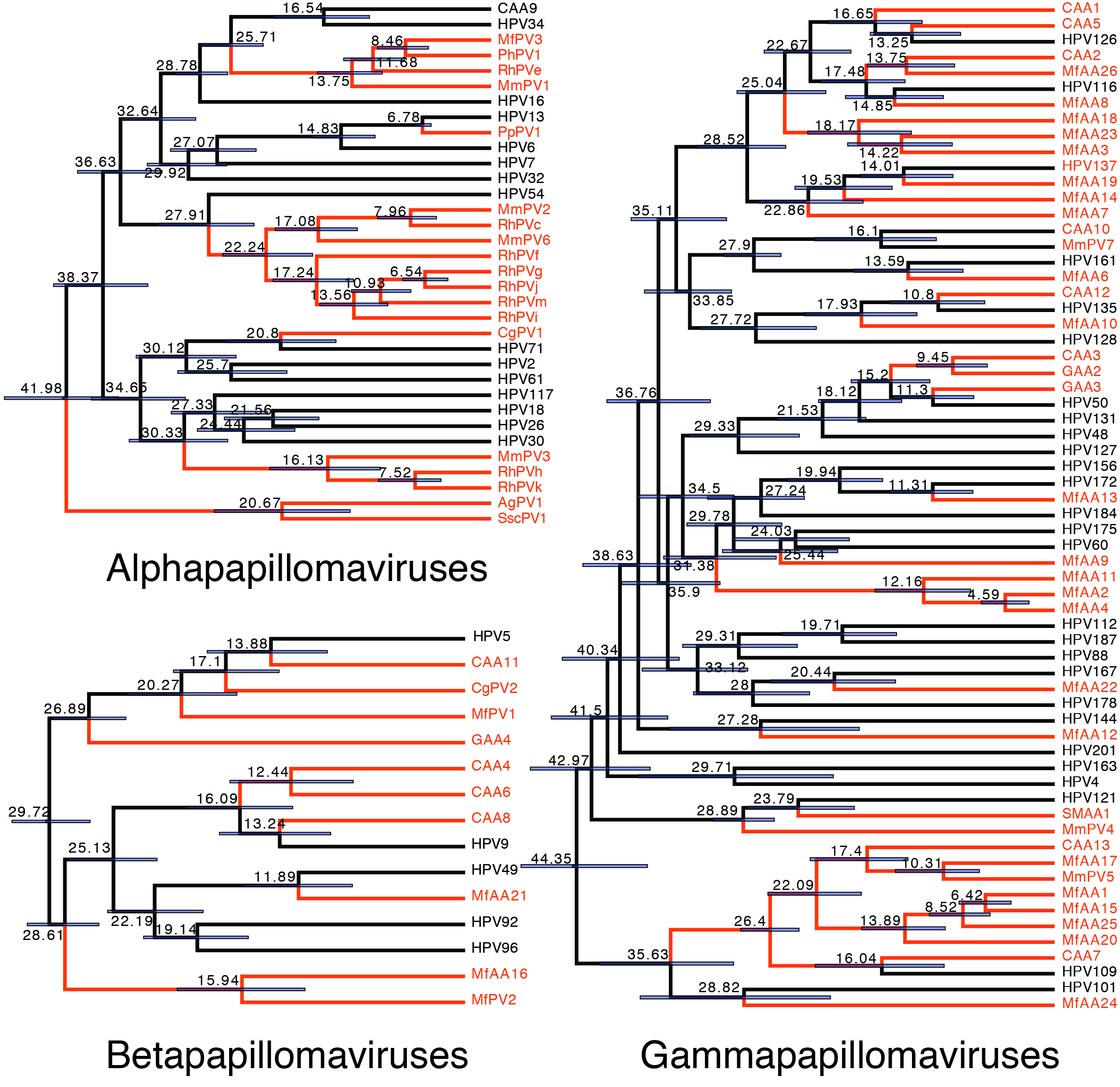

Supplement: FIGURE S7 — Divergence time estimation of Alphapapillomaviruses and Dyoomikronpapillomaviruses (A), Betapapillomaviruses (B), and Gammapapillomaviruses (C) inferred from partial L1 sequences by MY and FAP. A Bayesian MCMC method was used to estimate the divergence time of non-human primate PVs and HPVs from their most recent common ancestors. The branch lengths are proportional to the divergence times. The branches in orange refer to non-human primate papillomaviruses. The number on the nodes are the mean estimated divergence time in mya. The bar on the nodes represent the 95% HPD interval for the divergence times. [file Image_7.TIF]
